# Effect of Menstrual Cycle on Acute Intermittent Porphyria

**DOI:** 10.1177/2329048X17736170

**Published:** 2017-10-18

**Authors:** V. R. Souza Júnior, V. M. V. Lemos, I. M. L. Feitosa, R. Florencio, C. W. B. Correia, L. B. Correia Fontes, M. F. Hazin Costa, M. C. B. Correia

**Affiliations:** 1 Department of Clinical Medicine-Hematology, Universidade Federal de Pernambuco, Recife, Brazil; 2 The College of Medical, Veterinary and Life Sciences, University of Glasgow, Glasgow, United Kingdom; 3 Federal University of Pernambuco Society for Haematology, Recife, Brazil

**Keywords:** acute intermittent porphyria, anxiety, hyponatremia, behavior, pediatric

## Abstract

A 16-year-old female who was attended as an outpatient reported localized, acute abdominal pain with vomiting, symmetrical motor weakness, and burning sensation in both arms and legs. Her medical history showed irrational behavior, repeated admissions at the emergency units of many other reference hospitals, where she had been investigated for celiac disease and treated with analgesics for pain events. Her clinical condition remained unchanged despite the use of many oral analgesics. In those admissions, she showed dysautonomia, vomiting, and abdominal pain. Diagnosis investigation disclosed a notable serum hyponatremia (133.7 mEq/L). She was referred for endoscopy and the histopathological lesion of the antrum in the stomach did not show neoplastic lesions. Colonoscopy, pelvic magnetic resonance imaging (MRI), total abdominal computed tomography, and video laparoscopy were without significant abnormalities. Suspicion of acute intermittent porphyria was confirmed by quantitative urine porphobilinogen-level tests and genetic analysis. Patient was successfully treated with intravenous infusion of glucose and hemin therapy.

## Background

Acute intermittent porphyria is an inherited rare autosomal dominant disorder caused by mutation of low penetrance in the gene which is responsible for regulating heme synthesis by encoding porphobilinogen deaminase also named hydroxymethylbilane synthase. More than 300 porphobilinogen deaminase gene mutations have been recognized in acute intermittent porphyria, resulting in the loss of important enzymatic activity of porphobilinogen deaminase.^1^ The term porphyria comes from the Greek meaning purple. Porphyria’s biochemical framework is the overproduction and purple or eed porphyrins excretion.^2^

The Heme function includes diverse biological work such as electron transfer and oxygen transport. Heme synthesis occurs in all nucleated cells, but 2 production sites are important. The first is inside the bone marrow by erythroblast, where about 80% of heme are produced for hemoglobin use. The other is in hepatocytes, particularly the microsomal cytochrome P450 enzymes. The regulatory mechanisms in these 2 sites are different: erythroid heme synthesis depends on the availability of iron, while in the hepatic site it depends on the heme pool. The first heme synthesis process is the formation of 5-aminolevulinic acid which is catalyzed by an enzyme called 5-aminolevulinic acid synthase. The 2 isoforms of this enzyme are 5-aminolevulinic acid synthase 1 and 5-aminolevulinic acid synthase 2. 5-Aminolevulinic acid synthase 1 is regulated by intracellular hepatic heme pool which is the pharmaceutical effect of hemin in acute porphyria attacks.^3,4^

## Case History

A 16-year-old female from the northeastern region of Brazil who was attended as an outpatient at Instituto Materno Infantil de Pernambuco, a clinical reference hospital. She had localized, severe acute abdominal pain, mainly at epigastric and periumbilical region with profuse vomiting. Medical history showed repeated admissions at the emergency units of many other outpatient reference hospitals where she had been investigated for celiac disease and treated with oral analgesics. Her clinical condition remained unchanged despite the use of many oral analgesics. In those admissions, she progressed with dysautonomia, vomiting, acute abdominal pain, convulsions, symmetrical motor weakness, and a burning sensation in both arms and legs. Because of these monthly pain events, the patient abstained from school activities, jeopardizing her academic year. She informed that the first episodes of severe acute abdominal pain occurred when she was 12, during her menarche. She experienced episodes usually starting 10 days prior to the initiation of her menstrual period and usually persisted the entire menstrual cycle. Each pain episode was accompanied by reddish-colored urine excretion, nausea, and constipation. The woman reported she had irrational behavior and behavioral change. She often left home and forgot her destination or forgot why she had left, and sometimes she would leave without reason. She also has impulses to throw objects. Her medical history shows she suffered had anxiety, depression, and migraine. The patient has no sensitivity to the sunlight and no history of excessive alcoholic drinking, smoking, sexual intercourse, illicit drug usage, or food poisoning. Family history shows neither neurological diseases nor acute abdominal pain.

Physical examination revealed flat abdomen with mild tenderness on deep palpation mainly in the epigastric and periumbilical regions, normal bowel sounds, and no hepatosplenomegaly. Her urine culture test was negative. The celiac disease suspicion was ignored due to the normal levels of anti-transglutaminase immunoglobulin A, anti-endomysial immunoglobulin A, and anti-gliadin immunoglobulin A. Serum sodium levels were 133.7 mEq/L (normal: 135-145 mEq/L). Level of other serum electrolyte, glucose, urea, and creatinine were within the reference values. No abnormalities were observed in complete blood test or in liver function. Endoscopy and histopathological analysis of a lesion in the stomach antrum revealed no neoplastic lesion. Opaque enema had no megacolon signs. Colonoscopy, pelvic MRI, total abdominal computed tomography, and videolaparoscopy were without significant abnormalities.

Suspicion of acute intermittent porphyria was confirmed when the porphobilinogen urine test was strongly positive and quantitative urine porphobilinogen levels in 2 acute attacks were 5.51 and 31.5 mg/24 hours (normal: less than 2.4 mg/24 h). Table 1 shows results consistent with acute intermittent porphyria by measuring urinary porphobilinogen levels, which are increased during crises. She was successfully treated with intravenous infusion of glucose (300-400 g/d) and hemin therapy. Pain and nausea were controlled with morphine, tenoxicam, ondansetron, and paracetamol. Within a few days, the acute abdominal pain and other symptoms caused by acute porphyria attack resolved and the patient progressed normally. The patient’s porphyric crises is repeated monthly and ended when she takes hemin therapy.

**Table 1.** Laboratory Testing for Acute Intermittent Porphyria Diagnosis.

Component	Normal Condition mg/24 hours	On Acute Attack mg/24 hours	Reference Values mg/24 hours
Porphobilinogen	<0.9	31.5	<2.4

## Discussion

In Europe, the incidence of acute intermittent porphyria is approximately 5.4 people per million inhabitants, and its incidence is more common in countries such as United Kingdom and Sweden as reported by Elder et al.^5^ The majority of patients with acute intermittent porphyria are asymptomatic. Nevertheless, about 10% to 20% of acute intermittent porphyria heterozygote patients have acute attacks according to Balwani et al.^6^ The main clinical manifestations of acute attacks are abdominal pain in 90% of cases, generally associated with tetraparesis, urine color change (70%), constipation, hyponatremia in up to 40% of attacks, vomiting, peripheral neuropathy, mental confusion or convulsions, and psychiatric disorders (Table 2).^1,4,6-8^

**Table 2.** Common Signs and Symptoms on Acute Intermittent Porphyria^1,4,6–8^

Signs and Symptoms	Incidence, %
Neurological symptoms	
Nonspecific pain symptoms	50-70
Paresis	42-68
Respiratory paralysis	9-20
Muscle weakness	42-68
Psychiatric symptoms	40-58
Seizure	10-20
Gastrointestinal symptoms	
Abdominal pain	85-95
Vomit	43-88
Diarrhea	5-12
Constipation	48-84
Cardiovascular symptoms	
Tachycardia	28-80
Hypertension	36-54

The usual analgesics administered for abdominal pain had no benefit and may further worse the porphyric crisis possibly causing nausea and vomiting. In addition, other signs and symptoms such as muscle weakness, confusion, and hallucinations may lead to diagnosis according to clinical context. Including acute intermittent porphyria in the differential diagnosis of neurological, psychiatric, and gastroenterological disorders in seizures contributes to increased chance of correct diagnosis and appropriate treatment. In the case reported, the patient showed all of the main signs and symptoms, also including the most serious ones: abdominal pain, hyponatremia, and psychiatric with neurologic disorder.

The peripheral neuropathy is mainly motor and believed to result from axonal degeneration.^9^ Hyponatremia, present during patient’s painful crises, may be associated with acute porphyria attacks due to inappropriate secretion of antidiuretic hormone by the supposed supraoptic nuclei of damage to hypothalamus and might be due to diarrheal, vomiting, or renal sodium loss.^9-11^ Probably the main triggering factor for crisis is the menstrual cycle hormones. Progesterone is a porphyrins inductor and potent inducer of 5-aminolevulinic acid synthase 1, which is toxic to tissues when in high concentrations, and more phyrinogenic than oestrogens,^12^ explaining the more frequent attacks in luteal phase of this patient’s menstrual cycle. This also justifies why oral contraception inducts onsets and the preference for subdermal contraceptive implants.

Reduced intake of carbohydrate can also precipitate porphyrin attacks through indirect induction of 5-aminolevulinic acid synthase 1. Glucose downregulates 5-aminolevulinic acid synthase by affecting peroxisome proliferator-activated receptor gamma coactivator 1-alpha which is an important protein inductor of 5-aminolevulinic acid synthase 1 transcription.^13,14^ This low-carbohydrate diet could be for example due to illness and gastrointestinal upset. Alcoholic drinking is also implicated in the genesis of porphyria crisis because of alcohol is a direct 5-aminolevulinic acid synthase 1 inductor. Other triggers may include surgery, psychological disorder stress, tobacco, and infection as well as many unsafe medications (such as barbiturates, anticonvulsants, calcium channel blockers, some sedatives, antibiotics, antifungal, and hormones).^15^ However, not always attacks have a clearly identifiable trigger. All of these direct or indirect 5-aminolevulinic acid synthase 1 inductors act through increasing heme hepatic demand by consuming cytochrome P450 enzyme. Under normal conditions, the enzyme’s failure is not enough to initiate seizures. Triggers are needed to induce the symptoms and that is the reason as to why some people have mild symptoms only occasionally and other never present any symptoms. These patients are named as “latent” acute intermittent porphyria.^16^

Diagnosis is difficult not only due to nonspecific symptoms but also because of the complex interpretation of medical tests. Patients with acute intermittent porphyria show porphobilinogen urine test positive, and quantitative urine porphobilinogen level increased as shown in this case. Determination of erythrocyte deaminase levels in porphobilinogen is useful for diagnosis, but no change is found in up to 10% of cases. The gold standard for diagnosis is the screening for porphobilinogen deaminase gene mutations (test with 95% sensitivity and 100% specificity).^15^ More than 300 mutations were identified in the hydroxymethylbilane synthase gene (HYYYM) or porphobilinogen deaminase gene in acute intermittent porphyria.^17^

Treatment of seizures consists of hospitalization, suspension of porphyrinogenic drugs, and possible precipitating agents followed by infusion of hyperglycogenic diet and hematin which is a dark bluish or brownish pigment containing iron in the ferric state obtained by heme oxidation. Hematin inhibits porphyrin synthesis and stimulates globin synthesis.^18^

Although hematin is widely used to reduce the frequency of recurrent attacks in severely affected patients, complications such as difficulty to withdrawing treatment and iron overload due to long-term use can occur. Other possible treatment is using gonadotropin-releasing hormone that prevents ovulation, helping women with recurrent premenstrual attacks of porphyria, as reported in this case. The use of gonadotropin-releasing hormone therapy should be started on the first day of menstrual cycle in order to decrease the risk of porphiric attack precipitated by transitory ovarian stimulation.^1^ The hyperglycogenic diet is considered specific therapy for mild attacks which do not require hospitalization, because it downregulates hepatic 5-aminolevulinic acid synthase 1 which is the rate-limiting enzyme for heme biosynthesis in the liver. In this reported case, we associated the infusion of glucose with hematin which is more potent and used for severe attacks.^1,4,13^

The management of recurrent seizures requires the prescription of monthly infusions of 1 to 8 ampoules of heme arginate. Liver transplantation is reserved for patients with severe recurrent seizures, where drug management had no improvement over signs and symptoms.^19^ Patients with acute intermittent porphyria have good prognosis when properly treated; however, long-term complications such as hepatocellular carcinoma, chronic kidney insufficiency, and hypertension may occur.^17^

## Conclusion

The correct diagnosis and appropriate treatment of acute intermittent porphyria must be considered in differential diagnosis of any acute abdominal pain, neurological and psychiatric disorders not only to avoid unnecessary surgery such as nontherapeutic laparotomy but also to prevent the possibility of acute intermittent porphyria exacerbation using contraindicated drugs.
